# Plasma/Serum Proteomics based on Mass Spectrometry

**DOI:** 10.2174/0109298665286952240212053723

**Published:** 2024-04-25

**Authors:** Yiying Zhu

**Affiliations:** 1 Department of Chemistry, Tsinghua University, Beijing, China

**Keywords:** Proteomics, plasma/serum, mass spectrometry, diagnostic test, liquid biopsy, biomarker, clinical assay

## Abstract

Human blood is a window of physiology and disease. Examination of biomarkers in blood is a common clinical procedure, which can be informative in diagnosis and prognosis of diseases, and in evaluating treatment effectiveness. There is still a huge demand on new blood biomarkers and assays for precision medicine nowadays, therefore plasma/serum proteomics has attracted increasing attention in recent years. How to effectively proceed with the biomarker discovery and clinical diagnostic assay development is a question raised to researchers who are interested in this area. In this review, we comprehensively introduce the background and advancement of technologies for blood proteomics, with a focus on mass spectrometry (MS). Analyzing existing blood biomarkers and newly-built diagnostic assays based on MS can shed light on developing new biomarkers and analytical methods. We summarize various protein analytes in plasma/serum which include total proteome, protein post-translational modifications, and extracellular vesicles, focusing on their corresponding sample preparation methods for MS analysis. We propose screening multiple protein analytes in the same set of blood samples in order to increase success rate for biomarker discovery. We also review the trends of MS techniques for blood tests including sample preparation automation, and further provide our perspectives on their future directions.

## 1 INTRODUCTION

Liquid biopsy tests have a rich value in clinical settings, including diagnosis and prognosis of diseases, evaluation of treatment effectiveness, and risks. Blood specimens are relatively stable and can be easily accessed; therefore, they are the most widely used clinical liquid specimens. Three types of blood samples are collected, namely whole blood, plasma, and serum. Whole blood samples are usually used for determining blood cells: white blood cells, platelets, and red blood cells. Plasma and serum samples, which are the liquid portions of whole blood, are mostly used for clinical blood tests. Plasma is the upper part of the blood collected after the addition of anticoagulants (such as EDTA, heparin, and citrate) and centrifugation. Serum is the upper part of the blood after centrifugation when no anticoagulant is added to the blood. Fibrin blood clots, which are formed by coagulation factors combined with thrombin and fibrinogen, are precipitated and free from serum. Therefore, plasma and serum are not exactly the same in components; however, in most tests, they are interchangeable. There are different components in plasma and serum, in addition to cells in whole blood, including proteins, metabolites, nucleic acids, and other molecules. The majority of current blood biomarkers for routine diagnostic tests is proteins. However, the other components have limitations regarding their potential use as markers. For example, circulating tissue cells (CTC) released from tumors are used for monitoring cancer processes and treatment effectiveness but not for early diagnosis [1-3]. Circulating DNAs and mRNAs usually indicate probability risks [4, 5] and have been successfully applied in prenatal diagnosis [6-8]. Small-molecule metabolites, which have been studied extensively for clinical applications [9], can reveal patient phenotypes and biological mechanisms, including enzymatic and filtration processes. However, they are products of life processes and therefore only reveal limited information compared to proteins.

Current routine diagnostic tests are primarily based on blood protein biomarkers. They can be classified into three categories [10, 11]: 1) proteins that perform normal functions, such as human serum albumin (HSA) and apolipoproteins, which constitute the majority of the clinical test measures; 2) proteins released from tissues, such as aspartate aminotransferase (ASAT), alanine amino-transferase (ALAT), cardiac troponins, and tumor markers; and 3) signaling molecules and receptor ligands, such as small protein hormones (*e.g.*, insulin) and cytokines. In current medical practice, most clinical blood protein tests measure the level(s) of a single or a few protein(s) for decision-making. For example, diagnosis of acute events of troponin in myocardial infarction, thyroglobulin for detection of metastatic thyroid cancer recurrence after thyroid removal, and C-reactive protein for infection or inflammation [12]. Protein abundance ratios are used in some cases. Examples include the De Ritis ASAT/ALAT ratio to differentiate between the causes of liver disease [13] and the sFlt-1/PlGF ratio for the diagnosis of preeclampsia [14]. Modifications in specific proteins can also be used as markers. The core-fucosylated α-fetoprotein (AFP-L3) has been applied for the early diagnosis of hepatocellular carcinoma. Glycated hemoglobin A1c (HbA1c) levels serve as an indicator of long-term glucose exposure in the context of diabetes.

There is still a huge need for new blood tests to be developed for the early and companion diagnosis and monitoring of conditions and diseases. In addition to the techniques applied for protein identification and quantification in plasma and serum, the basics are also important for biomarker discovery and assay development. First, there is a consensus in the field of proteomics that it is critical to establish an organized integrated pipeline for blood biomarker discovery, validation, and assay development. The standardization of pre-analytics such as collection method, sample volume, storage temperature, processing, and analytics such as carryover, accuracy, precision, analytical sensitivity/specificity, and limit of detection/quantification has been extensively discussed in other review [11]. Plasma samples are mostly preferred to serum samples for biomarker studies, when the anticoagulant is tightly controlled. Plasma proteomes can be obtained reproducibly by using collection tubes preloaded with anticoagulants, and can delay sample preparation until 1 week at 4°C and until 25 freeze-thaw cycles [15-20]. Second, the study design is critical for blood proteomic research and assay development. The key gaps between disease diagnosis and intervention must be identified. Doctors typically recognize urgent clinical questions requiring blood tests. In addition, pharmaceutical companies that require the determination of a population to receive treatment for a particular drug or evaluation of the effectiveness of a drug require blood-companion diagnostic methods. It would be very beneficial for a public source, such as a website or national lab, to collect urgent problems and share them with the community. Third, sample sources are important, as clinical test development usually involves a large-scale study. At least 1500 samples are needed to validate a potential biomarker for further development of clinical diagnostic tests [21]. Hospitals, clinics, and clinical laboratories are the primary sources of human blood samples; biobanks are another source of bioliquids. For example, the UK Biobank has recruited over half a million UK participants and served researchers worldwide [22, 23]. The Shanghai Zhangjiang Biobank in China is projected to reach a storage capacity of 10 million human-derived samples, including plasma samples. Commercially available samples, such as those from the Plasma Services Group (Moorestown, NJ, USA), are used. Collecting and locating large amounts of blood samples under strict collection protocols with good records of patient information and classification remain challenging. For example, without proper handling, samples may contain proteins from blood cells, called hemolysis contamination, and blood cell proteins may falsely be taken to indicate the involvement of some pathways or identified as biomarkers; therefore, Greyer *et al.* profiled the total proteome of erythrocytes and platelets as an indicator of contamination from hemolysis in samples [24]. There are resources for the systematic classification of diseases that help differentiate patient cohorts, such as the International Statistical Classification of Diseases (https://icd.who.int/browse10/2019/en) and human phenome ontology, which can be used for precise phenotyping of patients [25].

## 2 PROTEOMICS TECHNOLOGIES

### 2.1 Affinity Reagents-based Technologies

Various proteomic technologies have emerged, which can be divided into affinity- and MS-based techniques. In this section, we briefly review the mechanisms of several popular methods based on affinity reagents antibodies and aptamers. Polyclonal antibodies are complex reagents produced by the animal immune system. In contrast, monoclonal antibodies bind to one antigen/epitope usually produced by hybridoma technology or as recombinant proteins. Traditional western blotting and enzyme-linked immunosorbent assays (ELISA) are low-throughput techniques. Proteins are spotted and bound onto a nitrocellulose membrane or coated onto a multi-well plate. Signal detection is based on chemiluminescent or fluorescent probes. New antibody-based technologies have increased the sample throughput and multiplexing ability based on novel protein spotting and detection methods, as shown in Figure (**1**). Proximity extension assay (PEA) by Olink (Uppsala, Sweden) is a special type of multiplexed antibody-based immunoassay. Two antibodies bind to one protein with the interaction domains in close proximity. Each antibody contains one DNA Oligonucleotide that can hybridize with each other. After the signal is transferred from the protein to DNA, it can be amplified by PCR and further detected by NGS or qPCR [26]. Reverse phase protein array (RPPA) is based on spotting proteins on nitrocellulose-coated glass slides and probing them with antibodies. This enables hundreds of microspots to be detected simultaneously on one slide [27]. Single-molecule array (SIMOA) bead technology from Quanterix (MA, USA) is highly sensitive. First, protein samples are added to antibody-coupled paramagnetic beads, followed by the addition of antibodies to form a sandwich, similar to standard sandwich ELISA. The sample is loaded into femtoliter-sized wells large enough for one bead, the so-called single-molecule array, and then visualized using enzyme-amplified florescence imaging [28]. The multi-analyte profiling (xMAP) protein assay (Luminex, TX, USA) is a bead-based immunoassay. A mixture of color-coded beads pre-coated with antibodies binds the analytes of interest and then conjugates biotinylated detection antibodies coupled with phycoerythrin (PE)-conjugated streptavidin. Multiplexed proteins are detected by a dual-laser flow-based detection instrument, where one laser classifies beads, determining the type of protein, and the other laser excites PE, indicating the number of analytes captured [29]. Aptamers, synthetic ligands made of DNA, RNA, or peptide, are another type of affinity reagents [30]. SomaScan is a representative aptamer-based protein assay based on the ssDNA SOMAmer reagent (SomaLogic, CO, USA). Bead-immobilized SOMAmers interact with target proteins and label them with biotin, after which the SOMAmers are photocleaved from the beads. After biotinylated proteins with SOMAmers are purified using monomeric avidin beads, SOMAmers are released and quantified by DNA-based technology [30]. These affinity-based technologies are discussed in more detail in other reviews [31-33].

### 2.2 MS-based Technologies

**Figure 2:**
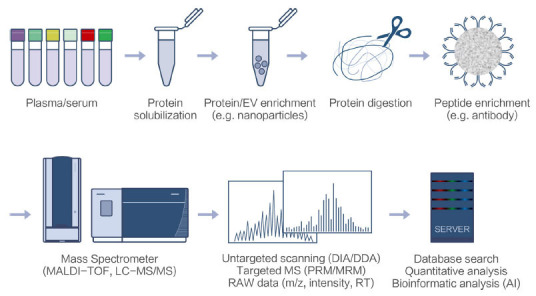
Schematic diagram of bottom-up MS-based proteomics.

MS is the only method that can detect proteins and their post-translational modifications (PTMs) without prior knowledge of the sample, whereas other affinity-based methods limit the detection of targets within the affinity reagent library. MS measures the mass-to-charge ratio of molecules, decodes amino acid sequences, and detects modifications in their side chains or termini. Here, we introduce the basic knowledge and terminology of MS and discuss the suitability of different MS technologies for clinical proteomics research and assay development. There are different types of MS instruments, which are categorized by their various ionization sources and mass analyzers. Matrix-assisted laser desorption/ionization (MALDI) and electrospray ionization (ESI) are the two most popular ionization sources. Surface-enhanced laser desorption/ionization (SELDI), a variant of MALDI, is valuable in clinical proteomics. The Food and Drug Administration (FDA)-approved MS-based diagnostic assay, OVA1, which combines five protein biomarkers for ovarian cancer, was developed using the SELDI-TOF platform [34, 35]. Time-of-flight (TOF) and orbitrap are the most popular high-resolution and high-mass-accuracy mass analyzers, whereas quadruple (Q) is the most popular mass filter. Recently, the analyzer Astral in Orbitrap Astral (Thermo Fisher Scientific, MA, USA) has emerged as a powerful analyzer with higher speed than TOF and orbitrap [36]. MALDI-TOF has already been widely applied in microorganism characterization in clinical settings by detecting abundant proteins in whole-cell lysates, such as ribosomes, which serve as biomarkers for microbial species [37-41]. With the assistance of artificial intelligence (AI) data interpretation, MALDI-TOF spectra can be used to predict antimicrobial drug resistance [42]. MALDI-TOF measures the mass-to-charge ratios of whole proteins in microbiological studies, which is called top-down proteomics. In contrast, bottom-up proteomics applies a workflow that employs proteases to cleave proteins, analyzes peptides by MS, and then composes protein information through the identified peptides (Figure **2**). Tandem mass spectrometry (MS/MS) is a strategy for deciphering the structures of molecules in which molecular ions (precursor ions) are scanned, selected, and fragmented, and fragment ions (product ions) are pictured as MS/MS spectra. MS/MS data can be used to identify peptides and their PTMs by utilizing search algorithms to match the mass-to-charge ratios of product ions between data from the spectra and in silico data predicted from a protein database. Tandem MS instruments equipped with MALDI, the so-called MALDI MS/MS, have become more and more popular [43]. Sometimes for convenience, MALDI TOF/TOF are referred to as MALDI-TOF which is regarded as a general name for the MS instrument equipped with MALDI and TOF. Tandem MS instruments equipped with ESI are usually coupled with liquid chromatography to separate the digested peptide mixture, which is the so-called LC-MS/MS where the word “ESI” is always omitted. This enables the measurement of thousands of proteins in one run within a short period [44]. Both MALDI-TOF and LC-MS/MS have a bright future in clinical settings. By comparing them, it is easy to reach a conclusion on their pros and cons and their suitability for particular projects. MALDI-TOF is easier and faster; however, it requires biomarkers to be abundant and prominent in the protein mixture. LC-MS/MS is more complex and slower but can detect many more proteins and decipher their peptide sequence structures. Therefore, LC-MS/MS is commonly used in biomarker discovery and protein modification studies. With recent developments, LC-MS/MS has become much faster and more stable; therefore, it has growing potential for the development of clinical assays. There are many variations and improvement in LC-MS/MS. For example, a scanning mode called data-independent acquisition (DIA), which differs from traditional data-dependent acquisition (DDA), collects fragments from all ions within defined m/z windows and provides the advantage of improved reproducibility, which is particularly valuable in large population studies. Isobaric labeling methodologies in LC-MS/MS, such as tandem mass tag (TMT), enable 18 channel multiplexed analysis and increase the peptide identification number per unit time [45]. Ion mobility technology coupled with MS, called field asymmetric ion mobility spectrometry (FAIMS) and trapped ion mobility spectrometry (TIMS), measures the sizes and shapes of analytes and provides another dimension of separation in addition to retention time, m/z, and peak intensities, thereby enhancing proteome coverage, and has been regularly applied in plasma/serum proteomics research [46-51]. In general, current clinical proteomics research reviewed here focuses mostly on LC-MS/MS and some on MALDI-TOF. We believe that LC-MS/MS will find application in clinical laboratories in the near future.

Targeted proteomics, in contrast to the aforementioned untargeted proteomics, monitors target peptides by sensitively deep scanning their precursor and product ions. Nature Methods chose targeted proteomics based on MS as the Method of choice in 2012 [52]. Multiple reaction monitoring (MRM) using triple quadrupole (QqQ) MS and parallel reaction monitoring (PRM) using quadruple Orbitrap/TOF instruments are targeted proteomic techniques. For decades, targeted MS has been used for the absolute quantification of small molecules in the pharmaceutical industry. QqQ and Q-Orbitrap or TOF are both (ESI)-LC-MS/MS types of MS instruments, whereas QqQ is the most popular one in targeted MS instruments because of its low cost and high speed. There are advanced targeted methods in advanced MS instruments based on spiked-in peptides to trigger the acquisition of targeted analytes that require no more retention time scheduling, such as TOMAHAQ [53], pseudo-PRM [54], and SureQuant [55]. The two advantages of targeted proteomics over conventional protein detection technology ELISA/western blotting and affinity-based methodologies are high specificity and multiplexing ability. High specificity comes from the direct measurement of peptides based on their mass-to-charge ratios, rather than signal transfer from affinity-binding reagents. For example, antithyroglobulin autoantibodies in the serum/plasma interfere with the thyroglobulin immunoassay, and MS provides a more accurate assay [56]. Another advantage of MS-based assays is that they enable multiple protein quantifications within one analysis, which greatly saves analytical time and reduces the required sample amount.

Enrichment or purification steps are critical for high-sensitivity analysis of low-abundance proteins or peptides. Coupling purification and enrichment with targeted MS combines the advantages of high sensitivity and specificity. Site-specific analysis of PTMs can be performed to enrich and analyze peptides carrying PTMs. Peptide enrichment is expected to be more sensitive than protein enrichment because it removes signal interference from other peptides of protein targets and their binding partners. We observed many examples of clinical assays developed using this method. Stable isotope standards and capture by anti-peptide antibodies (SISCAPA) by SISCAPA assay technologies (Washington D. C., USA) apply anti-peptide antibodies coupled to magnetic beads to enrich targeted peptides and then utilize MS to quantify peptides with the addition of stable isotope-labeled (SIL) peptides as internal standards [57, 58]. Antibody-conjugated nanodisk methods by NanoPin Technologies (Louisiana, USA) are applied to enrich target peptides from trypsin-digested serum samples to diagnose tuberculosis (TB) [59]. Clinical MS studies require good quality control for discovery and validation; therefore, internal standards and reference materials are important [60]. Generally, they are heavy isotope versions of the target peptides or recombinant proteins expressed with ^13^C-labeled arginine and lysine. For example, isotopically labeled IgG serves as the internal standard [61, 62]. Heavy isotope-labeled peptides are mostly used in targeted MS assays because of the relative simplicity of acquiring them [63]. The PQ500 reference peptides from Biognosis (Schlieren, Switzerland), which contain standard peptides for hundreds of plasma proteins, are good examples of commercialized heavy isotope-labeled plasma protein standard peptides for clinical studies [64].

Raw MS data must be processed to obtain meaningful information. First, raw data from LC-MS/MS contain several features, including mass-to-charge ratios (m/z), retention times (RT), and signal abundances of precursor and product ions, whereas MALDI-TOF data do not contain retention time features. Normally, the data go through a search algorithm that matches the mass-to-charge ratios of the product ions to those from the protein database to identify the peptides and their PTMs. Proteome Discoverer (Thermo Fisher Scientific, MA, USA), MaxQuant (https://www.maxquant.org) for DDA data, Spectronaut (Biognosis, Switzerland), and DIA-NN [65] for DIA data are all in this category. Protein glycosylation is the most complex PTM. Although other PTMs, such as phosphorylation, methylation, and acetylation, can be analyzed using the abovementioned software, glycosylation requires specific software for analysis. In an evaluation conducted by the community, including vendors and users of glycoproteomics informatics solutions [66] by comparing search results of the same files from serum glycopeptides, Protein Prospector [67] and Byonics (Protein Metrics, CA, USA) [68] stand out, providing high-performance software solutions for glycoproteomics, detecting hundreds of unique N-glycopeptides in serum. Publicly available software called pGlyco is another powerful tool for glycopeptide identification with robust quality control and false discovery rate (FDR) estimation, with unique features for glycan structure isomer differentiation [69, 70]. Glycopeptide quantification is mostly performed using label-free methods; other methods are discussed in more detail in another review [71]. The next part of the data analysis involves quantification after peptide identification. Most search algorithms contain a quantification module that is mainly based on the label-free method. Skyline is a quantitative software that can analyze data from various instruments and software. In Skyline, missing peaks or other errors in peak integration, which are factors that impair quantitation accuracy, can be trained or manually corrected. Efforts have been made to improve the peak integration of software for clinical data [72]. Finally, a table of proteins and their relative/absolute quantitation is sent for data mining; for example, statistical analyses for the identification of significant changes among different conditions and receiver operating characteristic (ROC) analysis, which shows the relationship between sensitivity and specificity for evaluating diagnostic performance. Recently, AI for proteomic data analysis has become increasingly popular; it finds rules among large amounts of data, although it is important to make the fitting more explainable because the mystery of data processing will prevent clinicians from using them [73]. Raw data from MALDI-TOF and LC-MS have been used for direct data analysis without database searching [42, 74]. It is expected that the advancement of data analysis ability and AI interpretation of raw data without manipulating the database search will become popular.

## 3 ANALYTES AND CORRESPONDING SAMPLE PREPARATION METHODS IN PLASMA/SERUM PROTEOMIC STUDIES

**Figure 3:**
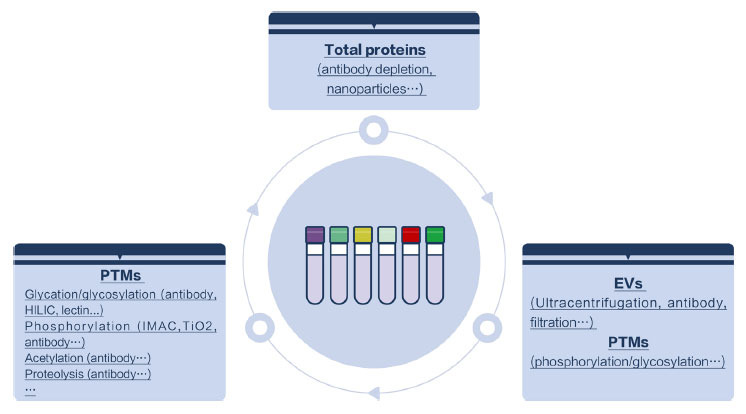
Analytes (total proteomes, PTMs, and EVs) in blood and their corresponding enrichment methods.

Discovery-based plasma/serum proteomics studies may target different analytes, from basic total proteome to protein PTMs to extracellular vesicles’ proteome (Figure **3**). We summarized their MS-based analytical methods, focusing on sample preparation methodologies and recent advances.

### 3.1 Total Proteome

Plasma/serum proteome analysis by MS faces significant difficulties because the concentrations of plasma/serum proteins vary up to the order of 10^12^, which is far beyond the dynamic range of MS. Reported concentrations of plasma proteins have been deposited in the blood proteomics database [75]. For example, the base level of interleukin-6 is approximately 5 pg/mL and the level of HSA is approximately 50 mg/mL. Fourteen of the most abundant proteins in the blood account for 95% of the total proteome in the blood, blocking the detection of the remaining low-abundance proteins. Currently, global MS has ng/mL sensitivity, whereas immunoassays have pg/mL sensitivity. To improve plasma proteome coverage, and before analysis by LC-MS/MS, plasma proteins are separated by an additional dimension, either off-line or on-line, such as gel electrophoresis, high-pH reversed-phase liquid chromate-graphy, size-exclusion chromatography, hydrophilic interaction chromatography, ion-exchange chromatography, and capillary electrophoresis [76]. With extensive fractionation and a long LC gradient, thousands of proteins can be detected in undepleted serum [77]. However, fractionation/separation inevitably requires time and leads to irreproducibility. The use of disposable or regenerable antibody columns/beads to conduct immunodepletion is a regular procedure for plasma/serum studies [78-81]. For example, Keshishian *et al.* applied IgY14 and SuperMix for abundant plasma protein depletion, combining isobaric labeling. An average of 4600 plasma proteins per sample were identified and 3400 proteins were quantified across 16 patients by a Q-Orbitrap MS instrument analysis [82]. However, the depletion step itself may cause problems, such as co-purification of proteins both specifically and non-specifically bound to abundant proteins and beads, making the procedure time-consuming, difficult to automate, and to perform with high throughput. Recently, a platform integrating nanoparticle protein corona to increase the depth of plasma proteome analysis in LC-MS/MS was developed and commercialized by Seer (MA, USA) [83-85]. With the aid of nanoparticle reagents, over a thousand proteins can be quickly identified and quantified in plasma using a simple procedure that reaches the proteome concentration range in which most FDA-approved plasma protein biomarkers lie [11].

### 3.2 Glycation and Glycosylation of Plasma/Serum Proteins

Protein glycation is a process in which glycans are nonenzymatically added to proteins. Advanced glycation end-products (AGEs) such as N-carboxymethyl-lysine (CML) and N-carboxyethyl-lysine (CEL) are produced by glycation [86]. AGEs not only accelerate the aging of the human body but also lead to the occurrence of many diseases [87-91]. Glycation of plasma proteins has a rich diagnostic value. The HbA1c test, which measures the percentage of glycated form in total hemoglobin, is a common blood test for the diagnosis of type 1 and type 2 diabetes. Efforts have been made to improve blood tests for diabetes by targeting other glycated blood biomarkers or applying new technologies, such as MS analysis of glycated end-product-modified peptides of HSA and beta-hemoglobin for better diagnostics [92]. Most human proteins are glycosylated by the enzymatic covalent addition of glycans. N-Linked and O-linked glycosylations are the most common types. Approximately 2600 N-linked glycosides and 1500 glycoproteins in human serum and plasma have been reported at the time of the review [93]. Glycosylation MS is difficult because of the wide variety and complex structures of glycans [94]. Glycans exhibit mass-to-charge ratios in MS Therefore, stereoisomers are usually classified into different categories. For example, glucose (Glc), galactose (Gal), and mannose (Man) are hexoses (Hex), and N-acetylglucosamine (GlcNAc) and N-acetylgalactosamine (GalNAc) are N-acetylhexosamines (HexNAc). In some cases, secondary and multistage MS incorporating the fragmentation of glycosidic bonds are used to resolve stereoisomers. For example, the relative abundances of oxonium ions can be used to differentiate between GlcNac and GalNac [95]. The structural isomers of glycans are recorded in some glycan databases, such as GlycomeDB [96, 97]. Search algorithms, such as pGlyco, for glycopeptides, can differentiate glycan structural isomers based on their different fragmentation types on glycosidic bonds. The fragmentation types in MS are important for glycoproteomics. Traditional collision-induced dissociation (CID), high-energy collision-induced dissociation (HCD), electrotransfer dissociation (ETD), and combinations of these, such as Stepped CID or EThcD, are methods that induce the breakage of amide and glycosidic bonds. Ultraviolet photodissociation (UVPD) is a promising dissociation method for a small-scale O-glycopeptide characterization [98]. By manipulating the energy of ion dissociation, studies are aiming to obtain a comprehensive mapping of peptides and glycans.

Analysis methods for protein glycosylation can release glycans and analyze the released sugar molecules or plain peptides [99] or directly analyze glycopeptides. Endoglycosidase cleaves glycans from the attached amino acid residues. PNGase is commonly used for the cleavage of N-glycans. A universal endo-O-glycosidase for O-glycans is not yet to be identified. Chemical beta-elimination approaches to release O-glycans are popular, but they can introduce side reactions, whereas some enzymes such as OpeRATOR and ImpaRATOR (Genovis, MA, USA) can cleave N-terminal of O-glycans attached serine and threonine [100, 101]. The direct analysis of glycopeptides is complicated; therefore, it is limited to simple proteins and requires manual validation. Recent technological advancements have enabled the analysis of glycopeptides globally. This review focuses on glycopeptide methodologies that have been applied in human plasma/serum proteomics [102-106]. Some of these have turned into blood diagnostic tests. For example, InterVenn Biosciences (CA, USA) established diagnostic tests based on the MS analysis of blood glycoproteins [107].

Enrichment of glycated peptides/glycopeptides prior to LC-MS/MS analysis is important because of their relatively low abundance. Borate/boronate affinity purification is popular for glycated peptides, and dozens to hundreds of glycated proteins have been identified in human serum and plasma [108-110]. Recently, AGE antibodies have been produced, providing an exciting way to analyze plasma protein AGEs [111]. Other reviews have discussed glycoproteomic enrichment strategies for MS analysis in detail [112, 113]. This review focuses on the methods that have already been applied to plasma samples. Lectins are proteins with a special affinity for glycans and provide specific enrichment methods for targeted glycan types [114, 115]. There is a large number of lectins available for glycoproteomics, such as concanavalin A (ConA) for mannose and glucose and wheat germ agglutinin (WGA) for N-acetylglucosamine and sialic acid [116]. Antibodies, as classical affinity reagents, provide methods for some types of glycosylated peptides, such as O-GlcNAc-modified peptides [117]. Other enrichment methods are available based on electrostatic and hydrophilic interactions with glycans, such as immobilized metal affinity chromatography (IMAC) for sialic acid containing glycopeptides [118-120], hydrophilic interaction chromatography (HILIC) [121-123], anion-exchange chromatography (AEC) [124], and electrostatic repulsion–hydrophilic interaction chromate-graphy (ERLIC) [125, 126]. Covalent addition based on chemical reactions is popular in glycoproteomics, such as hydrazide chemistry for O-GalNAc-modified proteins [127] and boronic acid-based chemistry for the universal enrichment of glycopeptides [128, 129], although they may induce side reactions and impact the results. To date, a high-throughput, simple glycopeptide enrichment strategy is still lacking or is not precise and widely accessible, although researchers are working on improving it [130]. In addition to the analysis of global glycosylation, it is valuable to analyze the glycosylation of target proteins to evaluate their impact on the activity or structure of the protein. In this case, if the protein is present in a complex mixture, an enrichment step for the target protein is required. For example, protein A beads for IgG or acid precipitation for AGP have been applied before the further purification of glycopeptides by HILIC chromatography or solid-phase extraction (SPE). Using these methods, researchers found higher fucosylation and sialylation of IgG subclasses in patients with pancreatitis than in patients with pancreatic cancer [61, 62], increased N-glycan branching, and lower sialylation of N-glycans on specific AGP glycosylation sites in individuals with hyperglycemia at high risk of diabetes [131].

### 3.3 Other PTMs of Plasma/Serum Proteins

Other protein PTMs in the plasma/serum can also serve as biomarkers [132, 133]; therefore, by analyzing them, we may find a connection between diseases or the effects of drug treatment and *in vitro* test methods for detecting them. Modified peptides are usually low-abundance; therefore, an enrichment step is necessary to analyze them by MS. Total phosphorylation of serine/threonine/tyrosine is commonly analyzed after enrichment of phosphorylated peptides by TiO_2_ or IMAC column/cartridge. Tyrosine phosphorylation accounts for a small percentage of total phosphorylation, and therefore requires an antibody for enrichment [134]. To date, many important PTMs have commercially available antibody affinity reagents [117, 135-138], which may be sequentially applied to samples to identify multiple PTMs in limited-amount samples. Gu *et al.* found abnormal lysine acetylation and arginine methylation in the sera of patients with acute myelogenous leukemia, breast cancer, and non-small-cell lung cancer [139]. As observed and surveyed by the author, this type of study is still rare, mainly because of the limitations of antibody reagents, which are only sold in kit format for tissue- or cell-based samples with limited immunoprecipitation numbers. With the incorporation of high-throughput and automated methods and the versatility of commercial antibodies in bulk format, they will become more popular in plasma/serum proteomics studies.

**Figure 4:**
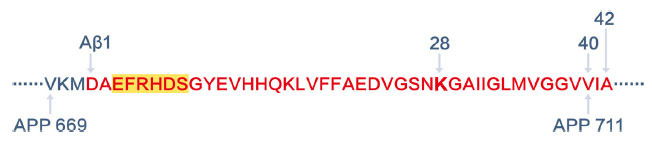
Cleavage sites of Aβ peptides.

Endogenous peptides in the plasma or serum owing to proteolysis may be considered as a special type of PTM. In this category, targeted MS analysis of amyloid-β (Aβ) peptides for diagnosis of Alzheimer’s disease is a good example. Aβ peptides are cleaved from the Aβ precursor and vary in length with differences of only a few amino acids (Figure **4**). Aβ deposition in the brain is the earliest pathological signature of Alzheimer’s disease. Using plasma biomarkers in predicting brain Aβ burden is beneficial for early detection and intervention of the disease, and also valuable for evaluating the effects of drugs that have potential for treating the disease. Nakamura *et al.* established a method incorporating immunoaffinity enrichment of plasma Aβ peptides and MALDI-TOF testing of the ratios of Aβ precursor protein (APP)_669-711_/Aβ_1-42_ and Aβ_1-40_/Aβ_1-42_, and their composites, to predict individual brain Aβ status [140]. They utilized magnetic bead bound anti-Aβ_1-16_ monoclonal antibody 6E10 (BioLegend, CA, USA) to enrich all three peptides, of which the affinity epitope lies within amino acids 3-8 of Aβ (EFRHDS) (Figure **4**). LC-MS/MS, which provides higher sensitivity and specificity than MALDI-TOF, can also be applied for plasma Aβ peptide testing. Kirmess *et al.* utilized HJ5.1 monoclonal antibody (C2N Diagnostics, MO, USA), which has affinity to amino acids 13-28 of Aβ, to extract plasma Aβ peptides, applied Lys-N to digest plasma Aβ peptides at lysine 28 (Figure **4**), and then employed LC-MS/MS to analyze the digested Aβ peptides [141]. These MS-based methods are proved to be more sensitive than ELISA test for Aβ peptides in human plasma.

Global peptidomic mapping is a method used to discover abnormal proteolytic activities. For example, a large number of circulating endogenous peptides in the plasma of patients with septic shock suggest significant systemic proteolysis, and further analysis of cleavage sites on the original proteins indicates a predominant role for serine proteases [142]. Clinical peptidomics, especially blood peptidomics based on MS, have been comprehensively reviewed in other studies [143, 144].

### 3.4 Extracellular Vesicles in Plasma/Serum

Much attention has been focused on the application of extracellular vesicles (EVs) in novel diagnostic and therapeutic strategies for various conditions such as cancers, neurodegenerative disorders, and cardiovascular diseases [145-147]. EVs are phospholipid bilayer membrane-enclosed structures containing RNAs, proteins, lipids, metabolites, and other molecules. Plasma EVs originate from circulating cells and endothelial cells owing to their constant interaction with blood flow, which can act as a mirror of cell status. They can generally be categorized into two main types according to their sizes and secretion pathways: large EVs, such as microvesicles (100-350 nm) released directly from the cell membrane, and small EVs, such as exosomes (30-150 nm) released by fusion of multivesicular bodies (MVBs) to the plasma membrane [148]. For biomarker discovery research, total proteomes in EVs are usually characterized. The PTMs in EVs may also serve as candidate biomarkers. For example, phosphoproteins in plasma EVs are candidate markers of breast cancer [149], and phosphoproteomic and N-glycoproteomic analyses can be sequentially performed in plasma-derived EVs [150].

The heterogeneity of EV enrichment methods may result in different populations and purities of EVs. Differential centrifugation methods remain the gold standard for EV purification, whereas the use of sucrose or iodixanol gradients in ultracentrifugation can eliminate nonspecific binding. Other purification methods include filtration, immunoaffinity purification, precipitation, and size-exclusion chromatography, which can be broadly applied in labs [151-154], together with several commercial kits already introduced to the market [155]. Because different purification methods may lead to substantially varying EV profiling results, the chosen method, including options for purchasing commercial kits, should be seriously considered when designing discovery and downstream validation experiments. To confirm the presence and purity of EVs after purification, microscopy and imaging technologies such as transmission electron microscopy (TEM) can be used. Characterization of protein markers in EVs can also be used. Proteins from the following classes can be used to demonstrate the presence of EVs: transmembrane or glycosylphosphatidylinositol (GPI)-anchored proteins associated with plasma membranes and/or endosomes (*e.g.* CD63, CD81) and cytosolic proteins (*e.g.* ALIX, HSC70) [154]. Immunoaffinity-based isolation strategies use antibody–antigen interactions to target specific populations of EVs. The immunocapture technique is considered the only one that can be directed toward the capture of a pure exosome population; therefore, it has been applied in many studies [156-159]. For example, tumor cell-derived exosomes (TEX) have emerged as key players in cancer progression. Capturing melanoma TEX with an anti-CSPG4 antibody and exosomes produced by T cells with an anti-CD3 antibody sequentially can isolate two subsets of patients with melanoma [160, 161]. Colon epithelial cell-specific A33 antibody was used to purify colorectal TEX [162].

## 4 TRENDS IN MS-BASED BLOOD PROTEOMICS

### 4.1 High-throughput and Automated Sample Prepa-ration

Proteomic sample preparation can be tedious, low-throughput, and easily introduces errors, which hinder the application of proteomic technology. Researchers have made significant efforts to achieve high-throughput and automated sample preparation, especially in plasma proteomics, and major improvements have been reviewed in detail [163, 164]. Generally, plasma proteomic sample preparation includes experimental steps of protein solubilization, digestion, depletion or enrichment, and desalting. The majority of these actions involve liquid handling steps with mixing and temperature control. Liquid handling robots for micro-chromatography-assisted sample preparation couple with multichannel pipettes, heating/shaking models, vacuum filtration, and magnetic bead stations. The replacement of manual pipettes with robotic liquid-handler systems increases analytical reproducibility. The SISCAPA methodology from SISCAPA assay technologies (Washington D. C., USA) incorporates immunoaffinity purification in sample preparation. Robots, such as the Bravo liquid handler (Agilent Technologies, CA, USA) [165, 166] and the KingFisher magnetic particle processor (Thermo Fisher Scientific, MA, USA) [58, 167] are suitable for immunoaffinity purification. Immunoaffinity purification is executed by pipetting/shaking, mixing the slurry with a magnetic station or cartridge/column/tips of protein A/G on top of pipetting, and slowly passing the solvent to the antibody-bound stationary phase to carry on the binding, washing, and separation steps. In addition to immunoaffinity purification, enrichment/separation steps using lectins and HILIC for glycosylated proteins or EVs can also be automated. Clean-up automation can be incorporated into the sample-introduction system of MS. Rapidfire (Agilent Technologies, CA, USA) is an integrated autosampler for ultrafast sample clean-up with automated SPE coupled to a QqQ MS [58]. The Evosep One LC system (Evosep Biosystems, Denmark) is a high-throughput automated uHPLC that incorporates automated SPE sample clean-up that can be matched to different types of MS instruments [168].

The challenge of using robots is that they require skilled technicians. Most of the time, there is a need for significant time to develop and implement protocols. Users are also required to spend time on routine maintenance and troubleshooting. Therefore, the simpler the steps are, the better. In addition to the costs of the instrument, consumables are usually vendor-specific, which leads to challenges in obtaining them and could be affected by supply shortages and high costs. Without automation, a high-throughput method with manual manipulation would also be interesting for the development of mini devices and associated consumables. The use of 96- or 384-well plates with manual or electronic multichannel pipettes may enable the processing of multiple samples for liquid handling. Tips or 96–364-well plates with C18 or other packing materials under centrifugation or pressure systems enable multisample enrichment and desalting. The 96-well plate format is the most common for proteomics when dealing with sample volumes of a few to two hundred microliter levels [169]. We look forward to the same platforms that could be used in both biomarker and clinical assay developments, which could eliminate the development of immunoassays for IVD and significantly speed up the development of new clinical assays.

### 4.2 Fast and Miniature MS

MS has become much faster in recent years and provides the ability to perform deep proteome analysis for biomarker discovery. Recently released Orbitrap Astral MS instrument (Thermo Fisher Scientific, MA, USA) analyzed eight thousand cellular proteins within 8 min, significantly increasing the throughput of MS analysis [36]. QqQ and MALDI-TOF are the most commonly used MS instruments for protein assays in clinics and pharmaceutical companies [43, 170, 171]. There is an increasing need for the application of MS in clinical research and assay validation. In this case, their large size, high requirement for vacuum and electronic systems, and high cost prevent their broader application. Compact, miniature, or even portable MS with reduced size and requirements for vacuum and electronic systems is more favorable. Efforts on miniature MS have been made for a long time, beginning in the 1970s, and advancements in these technologies have continued, with many new mini MS being commercialized [172]. Studies have mainly focused on small molecules, with some pioneering studies on proteins and peptides. Zhai *et al.* demonstrated direct analysis of pure peptides and proteins using laser spray ionization miniature MS [173]. Chiang *et al.* detected peptides from the MET protein in a complex SKBR3 cell lysate after immunoaffinity enrichment of the MET peptides using a miniature MS named Mini β (PURSPEC Technologies, Beijing, China) equipped with a discontinuous atmospheric pressure interface (DAPI), a pulsed nanoESI source, and dual linear ion traps [174]. Future miniature MS may act like and replace the western blot imager as an easy benchtop, daily-use protein characterization method.

### 4.3 Increased Demand for Research Collaboration

Current clinical laboratory test research mainly involves collaborations between doctors using blood sample resources and researchers using facility resources. Research centers or scientific service companies house good scientific instruments, researchers, and technicians. Therefore, research on bioliquid specimens is largely dependent on who is known in the clinic. It would be significantly helpful if a nationwide medical association could develop a list of clinical diagnostic questions according to needs and urgency, as well as organize blood collection from different medical centers. Currently, some large hospitals with sufficient funding have established facility cores and have recruited researchers specializing in proteomics and data processing. However, to date, most clinical researchers do not have a core proteomics facility in their workplace. It would be helpful for core facilities in the nation to have an organization to share information on the capability and availability of their instruments. The Core Technologies for Life Sciences (CTLS), founded in Europe, and the Association of Biomolecular Resource Facilities (ABRF), founded in the US, are organizations that bring together scientists, technical staff, and administrative staff working in shared resource laboratories. It would be interesting and beneficial to establish a similar association in China.

## CONCLUSION

MS is a powerful and valuable technique for measuring plasma and serum proteins and their PTMs. Targeted MS combined with immunoaffinity enrichment provides a highly specific and sensitive method for the development of clinical blood tests. Total proteome, protein PTMs, and EVs in the plasma/serum are different analytes for blood proteomics studies using different sample preparation methods. It would be helpful if all the sample preparation steps could be incorporated into one platform and automated. High-resolution, high mass accuracy LC-MS/MS has become increasingly important in clinical applications. For biomarker discovery research, sophisticated MS is needed, whereas, for assay development, small, robust MS at low cost has advantages. A robust and comprehensive pipeline for biomarker discovery, validation, and assay development, especially for various protein targets in the plasma/serum, will greatly help in the emergence of new blood tests, which will ultimately help realize precision and personalized medicine. With increasingly broad collaboration between people in clinical settings and MS cores, together with the advancements in these technologies, new blood protein tests are expected to develop rapidly in the near future.

## Figures and Tables

**Figure 1:**
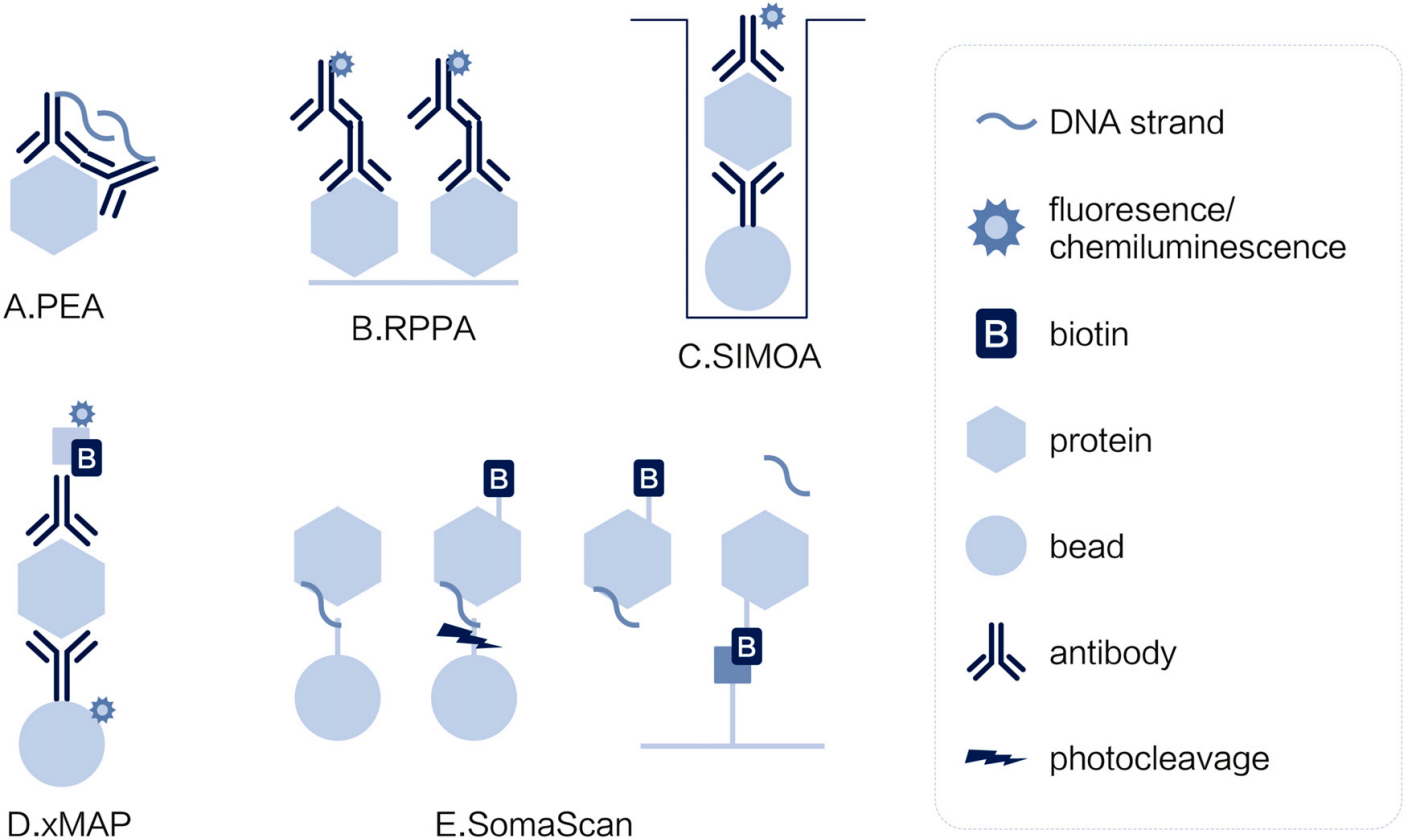
Schematic diagram of different affinity-based proteomic technologies.

## LIST OF ABBREVIATIONS

MS: Mass Spectrometry
MS: Mass Spectrometer
CTC: Circulating Tissue Cells
HSA: Human Serum Albumin
ASAT: Aspartate Aminotransferase
ALAT: Alanine Aminotransferase
HbA1c: Glycated Hemoglobin
AFP-L3: Core-fucosylated Α-fetoprotein
ELISA: Enzyme-linked Immunosorbent Assays
PEA: Proximity Extension Assay
RPPA: Reverse Phase Protein Array
SIMOA: Single-molecule Array
xMAP: Multi-analyte Profiling
PE: Phycoerythrin
PTMs: Post-translational Modifications
MALDI: Matrix-assisted Laser Desorption/Ionization
ESI: Electrospray Ionization
SELDI: Surface-enhanced Laser Desorption/Ionization
FDA: Food and Drug Administration
TOF: Time-of-Flight
Q: Quadruple
AI: Artificial Intelligence
MS/MS: Tandem Mass Spectrometry
DIA: Data-independent Acquisition
DDA: Data-dependent Acquisition
TMT: Tandem Mass Tag
FAIMS: Field Asymmetric Ion Mobility Spectrometry
TIMS: Trapped Ion Mobility Spectrometry
MRM: Multiple Reaction Monitoring
QqQ: Triple Quadrupole
PRM: Parallel Reaction Monitoring
SISCAPA: Stable Isotope Standards and Capture by Anti-Peptide Antibodies
SIL: Stable Isotope-labeled Peptides
m/z: Mass-to-Charge Ratios
RT: Retention Times
FDR: False Discovery Rate
ROC: Receiver Operating Characteristic
AGEs: Advanced Glycation End-products
CML: N-carboxymethyl-lysine
CEL: N-carboxyethyl-lysine
Glc: Glucose
Gal: Galactose
Man: Mannose
Hex: Hexoses
GlcNAc: N-acetylglucosamine
GalNAc: N-acetylgalactosamine
HexNAc: N-acetylhexosamines
CID: Collision-induced Dissociation
HCD: High-energy Collision-induced Dissociation
ETD: Electrotransfer Dissociation
ConA: Concanavalin A
WGA: Wheat Germ Agglutinin
IMAC: Immobilized Metal Affinity Chromatography
HILIC: Hydrophilic Interaction Chromatography
AEC: Anion-exchange Chromatography
ERLIC: Electrostatic Repulsion–hydrophilic Interaction Chromatography
SPE: Solid-phase Extraction
Aβ: Amyloid-Β
APP: Aβ Precursor Protein
EVs: Extracellular Vesicles
MVBs: Multivesicular Bodies
TEM: Transmission Electron Microscopy
GPI: Glycosylphosphatidylinositol
TEX: Tumor Cell-derived Exosomes
CTLS: Core Technologies for Life Sciences
ABRF: Association of Biomolecular Resource Facilities
